# Thioredoxin-1 mediates neuroprotection of Schisanhenol against MPP^+^-induced apoptosis via suppression of ASK1-P38-NF-κB pathway in SH-SY5Y cells

**DOI:** 10.1038/s41598-021-01000-3

**Published:** 2021-11-03

**Authors:** Hongyan Yang, Libo Li, Yu Jiao, Yuanliang Zhang, Yuhua Wang, Kunjie Zhu, Chao Sun

**Affiliations:** 1 Department of Pharmacy, Qiqihar Medical University, Qiqihar, China; 2 School of Mental Health, Qiqihar Medical University, 333 Bukui Street, Jianhua District, Qiqihar, 161006 China; 3 State Key Laboratory of Chemical Biology and Drug Discovery, Department of Applied Biology and Chemical Technology, Hong Kong Polytechnic University, Hong Kong, China; 4 Basic Medicine School, Qiqihar Medical University, Qiqihar, China

**Keywords:** Parkinson's disease, Parkinson's disease, Parkinson's disease

## Abstract

Oxidative stress-induced dopaminergic neuronal loss and apoptosis play a crucial role in the pathogenesis of Parkinson’s disease (PD), and as a vital antioxidant protein, thioredoxin (Trx) exerts neuroprotection against PD. In this study, we investigated the effect of Schisanhenol (Sal), an active component from a traditional Chinese herb *Schisandra rubriflora* (Franch.), on MPP^+^-induced apoptosis and its association with thioredoxin-1 (Trx1) in SH-SY5Y cells. The protein levels of Trx1 and apoptosis-related proteins were detected by Western blot, the expression of Trx1 mRNA by real time qPCR, and apoptosis was detected by fluorescence microscopy and flow cytometry. Pretreatment with Sal (1 µM, 10 µM, and 50 µM) dose-dependently ameliorated MPP^+^-induced neuronal injury, confirmed by the improvement of the viability and morphological changes. Sal decreased the apoptosis rate of cells, suppressed the production of DNA ladder and sub-G1 peak, inhibited the Caspase-3 activity and the expression of apoptosis-related proteins. Sal enhanced the expression of Trx1 both in the protein and mRNA levels. However, the Trx1 inhibitor PX-12 suppressed the protective effects of Sal. In addition, Sal inhibited NF-κB translocation and activation. These results suggest that Sal has a protective effect against MPP^+^-induced apoptosis in SH-SY5Y cells via up-regulation of Trx1 expression and suppression of ASK1-P38-NF-κB pathway.

## Introduction

Parkinson’s disease (PD) is a neurodegenerative disease characterized by progressive motor disorder and loss of dopaminergic neurons in the substantia nigra pars compacta^1,2^. Current therapies only ameliorate symptoms without rooting out progressive neuronal loss^3^. Although the cause of PD remains unclear, oxidative stress is acknowledged as one of the crucial factors in the pathogenesis of PD^4^. Oxidative stress originates from reactive oxygen species (ROS), which is mainly from the metabolism of dopamine and mitochondrial dysfunction^5,6^. Thus, inhibiting oxidative stress is proposed to be a potential therapeutic strategy for PD, and seeking neuroprotective agents from anti-oxidants is an ideal approach to prevent or cure PD.

Thioredoxin (Trx) is an essential component of the Trx system which plays crucial roles in regulating multiple cellular redox signaling pathways^7^, modulating transcription factors, and inhibiting apoptosis^8^. In mammalian cells there are two isoforms of Trx, Thioredoxin-1 (Trx1) and Thioredoxin-2 (Trx2). Trx1 is mainly located in cytosol, also translocates to the nucleus and can be secreted from cells under certain circumstances, whereas Trx2 is only located in mitochondria^9^. In the present study, we focused on Trx1. Trx1 is a 12-kDa ubiquitous protein with disulfide-reducing activity, and a pivotal antioxidant protein that protects cells from oxidative stress. Highly expressed in the central nervous system of mammals, Trx1 is an important regulator of neuroprotection^10^ to control the pathogenesis of PD. Accumulating evidence has proven the neuroprotective effect of Trx1 on neurons against PD-inducing toxicants. DJ-1-induced Trx1 protected SH-SY5Y cells and substantia nigra of mice from oxidative stress^11^. Trx attenuated 1-Methyl-4-phenylpyridinium iodide (MPP^+^)-induced neurotoxicity in PC12 cells^12^, and resisted apoptosis of dopaminergic neurons in 1-methyl-4-phenyl-1, 2, 3, 6-tetrahydropyridine (MPTP) exposed mice^13^. Trx exerted neuroprotection against oxidative stress-induced apoptosis in SH-SY5Y cells^14^, decreased the formation of α-Synuclein inclusions^15^. These results suggest that Trx or Trx inducer is a potential new therapeutic agent for PD.

**Figure 1:**
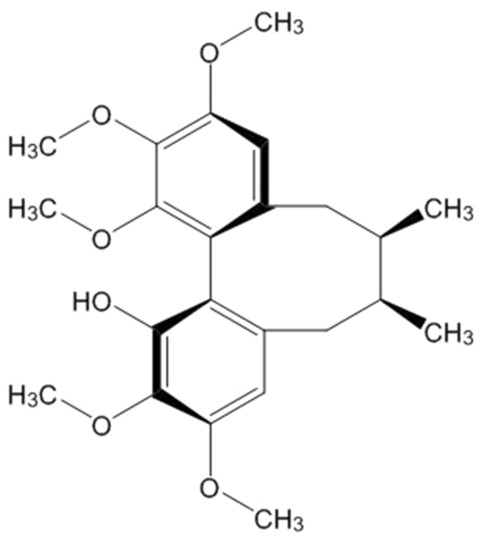
Chemical structure of Schisanhenol.

Schisanhenol (Sal; chemical structure shown in Fig. 1) is an active component from the fruit of a traditional Chinese herb *Schisandra rubriflora* (Franch.). Sal has significant anti-oxidant activity and neuroprotective effect. In vitro, Sal scavenged free radical^16^, inhibited peroxidation in rat liver microsomes^17^, protected against Fe^2+^-Cys-induced injury in rat cerebral mitochondria and synaptosomes^18^. Sal also inhibited the production of ROS and the oxidation of human low density lipoprotein^19^, and attenuated ox-LDL-induced apoptosis and ROS generation in bovine aorta endothelial cells^20^. In vivo, Sal inhibited the peroxidation of brain mitochondria and membrane in rats^21^, and protected against ROS-induced injury of spleen lymphocytes in mice^22^. In addition, Sal can distribute in brain through blood–brain barrier after oral administration in mice^23^. Therefore, it is worthy of further investigation on the neuroprotection and mechanism of Sal for neurodegenerative diseases, such as PD.

In the present study, we aimed to investigate the neuroprotective effect of Sal against MPP^+^-induced apoptosis, as well as explore the underlying mechanisms of Sal in human neuroblastoma SH-SY5Y cells, to elucidate whether Trx1 is one of the molecular targets of Sal.

## Results

### Sal increases cell viability in MPP^+^-treated SH-SY5Y cells

**Figure 2:**
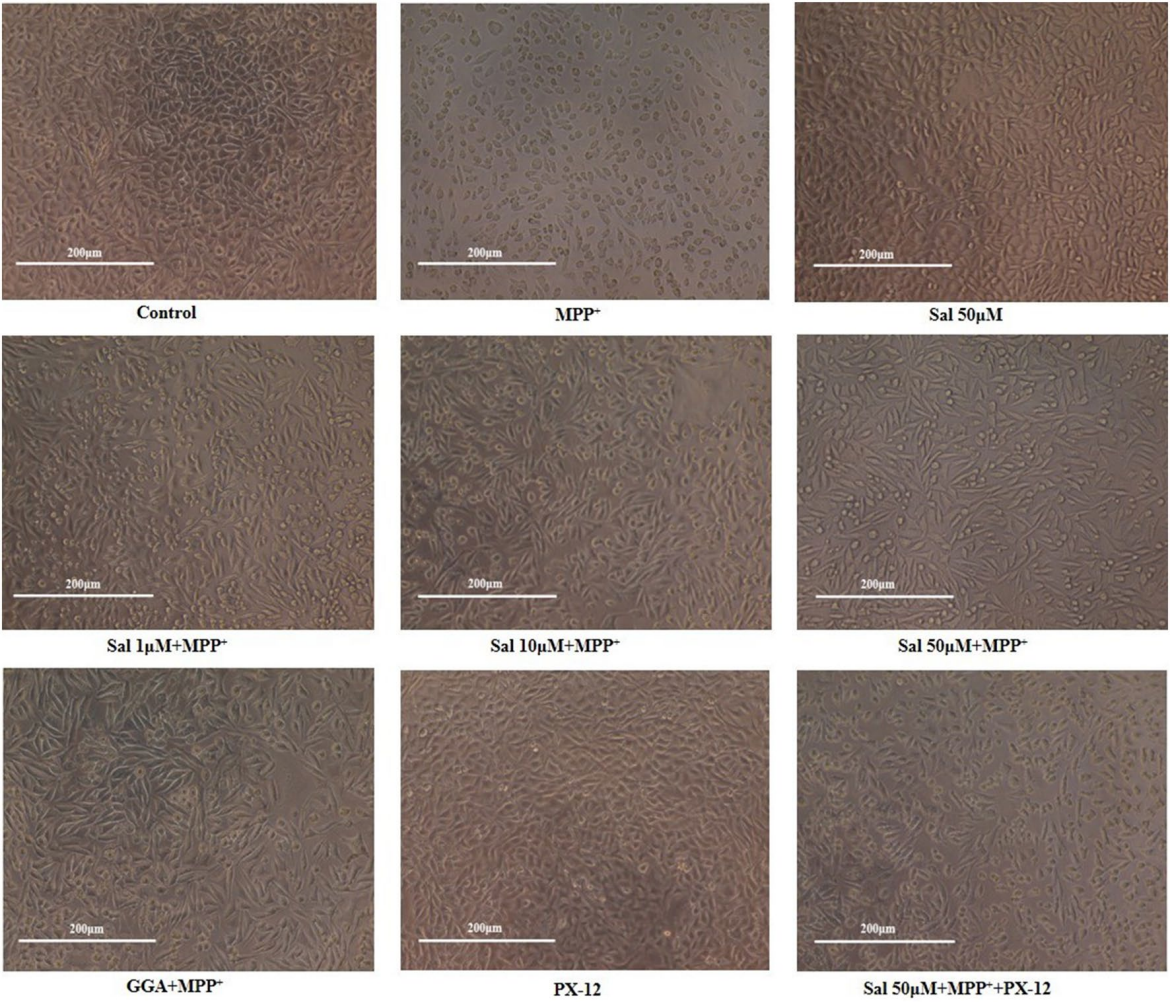
Protective effect of Schisanhenol (Sal) on morphology of SH-SY5Y cells treated with MPP^+^ via Trx1. Cells were pre-incubated in various doses of Sal (1 µM, 10 µM, and 50 µM) for 24 h before exposed to MPP^+^ (500 μΜ) for 48 h. Images were obtained with inverted phase-contrast microscope (100 ×). Representative images from six separate experiments were shown. Each experiment was conducted in triplicate. Scale bar, 200 μm.

To evaluate the toxicity of MPP^+^, SH-SY5Y cells were treated with different concentrations of MPP^+^ (0.1 mM, 0.3 mM, 0.5 mM, 1 mM, and 5 mM) for different time (24 h, 36 h, and 48 h); cell viability was subsequently measured using the MTT assay Kit. The results showed that MPP^+^ significantly decreased cell viability in time- and dose-dependent manner (see Supplementary Fig. S1 online). 500 μΜ MPP^+^ exposure for 48 h was used for subsequent experiments. To assess the neuroprotective effect of Sal, SH-SY5Y cells were pre-incubated in various doses of Sal (1 µM, 10 µM, and 50 µM) for 24 h before exposed to MPP^+^ (500 μΜ) for 48 h. Sal increased the cell viability of SH-SY5Y cells treated with MPP^+^ in a dose-dependent manner. Moreover, Sal did not show cytotoxic effects on SH-SY5Y cells even at 50 µM (Table 1). Morphological alterations of the cells were also observed under an inverted phase-contrast microscope; this result was consistent with the MTT results. The cells in the control group were polygonal and grew well with multiple, short, and fine cell processes. By contrast, MPP^+^ resulted in clear damage of the cells, with the neurites either retracted or disappeared. Approximately half of the cells treated with MPP^+^ were fusiform or spherical, and were floating instead of adhering, with low refraction. Sal significantly improved cell morphology in a dose-dependent manner at 1 μM, 10 μM, and 50 μM (Fig. 2). The protection of Sal at 50 μM was better than that of Geranylgeranylacetone (GGA), which is a Trx inducer and a protective agent against MPP^+^^12,24^. However, the protective effect of Sal was inhibited by co-treated with 2-[(1-Methylpropyl) dithio]-1H-imidazole (PX-12), a Trx1 inhibitor^25,26^. Sal 50 μM per se did not exhibit significant effects on cell viability and neither did PX-12. These results indicated that Sal exhibited a remarkable neuroprotective effect with high safety.

**Table 1:** Protective effect of Schisanhenol on viability in MPP^+^-treated SH-SY5Y cells via Trx1.

Group	Cell viability (%)effective rate
Control	100.0 ± 2.22
MPP^+^	49.23 ± 4.57**
Sal 1 μM + MPP^+^	70.66 ± 5.42^# #^
Sal 10 μM + MPP^+^	81.25 ± 3.97^# #^
Sal 50 μM + MPP^+^	94.33 ± 3.28^# #^
Sal 50 μM + MPP^+^ + PX-12	67.85 ± 3.96^&&^
GGA + MPP^+^	84.59 ± 2.45^# #^
PX-12	99.76 ± 4.70
Sal 50 μM	99.70 ± 3.56

SH-SY5Y cells were pre-incubated with Sal (1 µM, 10 µM, and 50 µM) for 24 h and then were exposed to MPP^+^ (500 μΜ) for 48 h. Cell viability was determined by MTT assay. Data were expressed as mean ± SD from six separate experiments, and each experiment was performed in triplicate. **P < 0.01 vs Control group; ^##^*P* < 0.01 vs MPP^+^ group; ^&&^P < 0.01 vs Sal 50 μM + MPP^+^ group.

### Sal suppresses MPP^+^-induced apoptosis in SH-SY5Y cells

**Figure 3:**
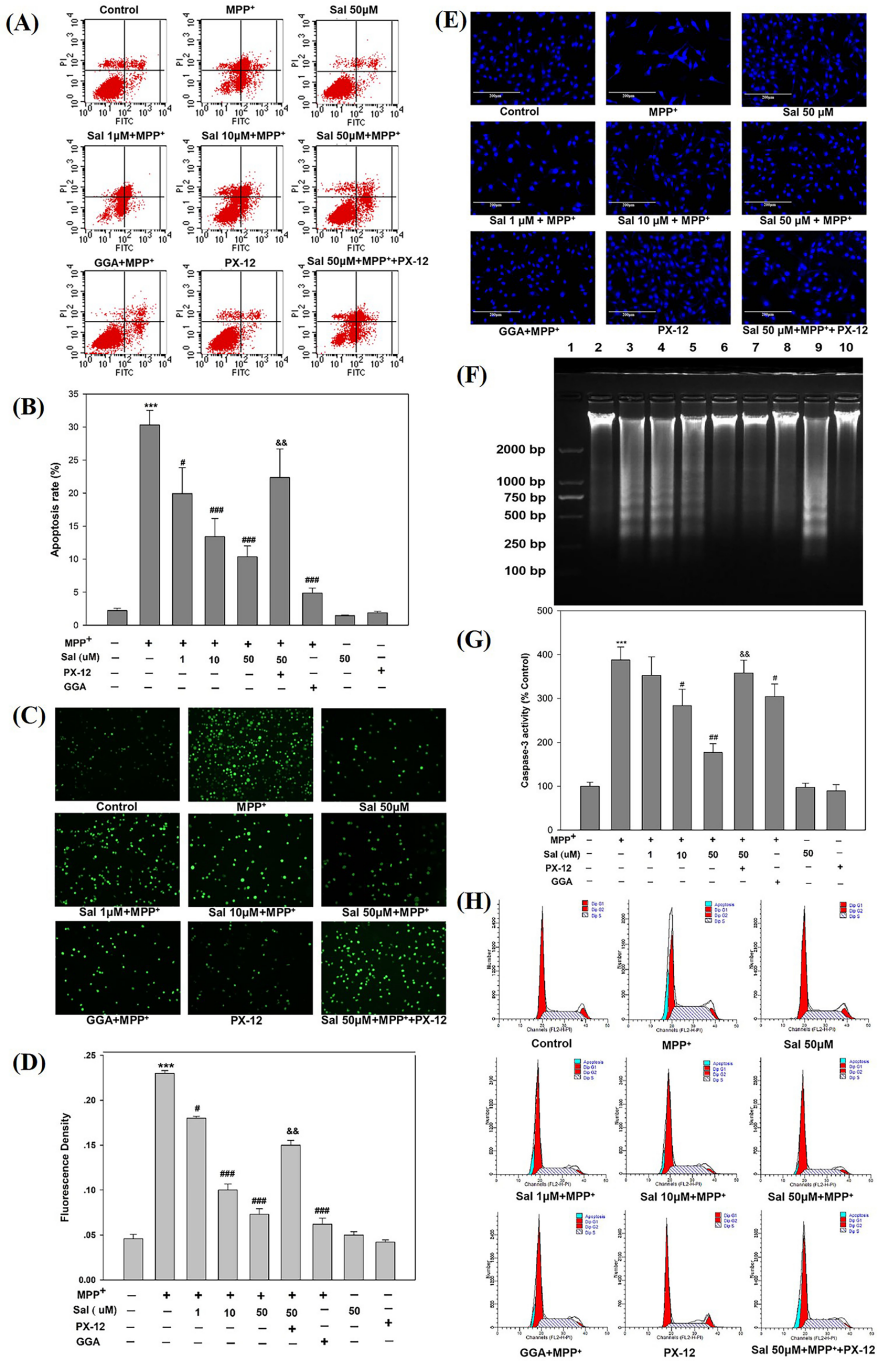
Schisanhenol attenuates apoptosis in SH-SY5Y cells treated with MPP^+^ via Trx1. Cells were pre-treated with Sal (1 µM, 10 µM, and 50 µM) for 24 h before the addition of 500 μΜ MPP^+^. (**A**) Apoptosis was evaluated by Annexin V-FITC/PI staining via flow cytometry, and a representative plot from six separate experiments was shown; (**B**) Cell apoptosis rate of (**A**) was calculated. (**C**) Cell apoptosis was also detected under florescent microscope after (**A**), and a representative image of six independent experiments was shown; (**D**) The fluorescence density of (**C**) was calculated; (**E**) Apoptotic nuclear morphology was visualized by DNA staining with Hoechst 33,342 (200 ×), and a representative image from six separate experiments was shown; (**F**) Cells were harvested and fragmented DNA was extracted and analyzed by agarose gel electrophoresis. Lane 1- DNA marker; Lane 2- Control; Lane 3- MPP^+^ ; Lane 4- Sal 1 μM + MPP^+^; Lane 5- Sal 10 μM + MPP^+^; Lane 6- Sal 50 μM + MPP^+^; Lane 7- GGA + MPP^+^ ; Lane 8- PX-12; Lane 9- Sal 50 μM + MPP^+^  + PX-12; Lane 10- Sal 50 μM. A representative image from six independent experiments was shown; (**G**) Caspase-3 activity was determined using colorimetrical assay with six independent experiments; (**H**) Cell cycle was detected by flow cytometric assay. A representative plot of six separate experiments was shown. ***P < 0.001 vs Control group; ^#^*P* < 0.05, ^##^*P* < 0.01, ^###^*P* < 0.001 vs MPP^+^ group; ^&&^*P* < 0.01 vs Sal 50 μM + MPP^+^ group. The above experiments were performed in triplicate each.

The effect of Sal on cell apoptosis was investigated by flow cytometry and fluorescence imaging using the Annexin V-FITC/PI staining. Cytometry assay showed that MPP^+^ increased the apoptosis rate of SH-SY5Y cells, and Sal pretreatment significantly decreased the MPP^+^-induced apoptosis rate (Fig. 3A, B), this result was in accordance with that of the fluorescence density (Fig. 3C, D). Meanwhile, Hoechst 33,342 staining was also used to detect the apoptotic cells. After being stained with Hoechst 33,342, the cultured SH-SY5Y cells were observed under a fluorescence microscope. Figure 3E shows that MPP^+^ exposure led to heterogeneous staining, nucleus condensation, and fragmentation, which were few in the control group. However, in the presence of Sal, the number of apoptotic cells was markedly decreased compared with the cells in MPP^+^ model group. These results revealed that Sal suppressed cell apoptosis induced by MPP^+^ in SH-SY5Y cells. Trx1 inhibitor PX-12 attenuated the above effects of Sal.

### Sal inhibits MPP^+^-induced DNA fragmentation in SH-SY5Y cells

According to the description in the Methods section, agarose gel electrophoresis was performed to investigate the effect of Sal on MPP^+^-induced DNA fragmentation in SH-SY5Y cells. The results showed that exposure to MPP^+^ for 48 h produced DNA fragmentation in SH-SY5Y cells, as indicated by DNA ladder. By contrast, pretreatment with Sal substantially reduced the degree of DNA fragmentation and this effect was also attenuated by PX-12, the Trx1 inhibitor (Fig. 3F).

### Sal decreases the Caspase-3 activity in SH-SY5Y cells treated with MPP^+^

We also examined the activity of Caspases-3 using colorimetrical assay to determine the effect of Sal on MPP^+^-induced apoptosis. Compared with the control group, the activity of Caspase-3 was significantly increased in MPP^+^ model group. However, Sal decreased the activity of Caspase-3 at the dose of 10 μM and 50 μM, and the Trx1 inhibitor PX-12 attenuated this effect of Sal (Fig. 3G).

### Sal affects the cell cycle in MPP^+^-treated SH-SY5Y cells

To analyze the effect of Sal on the cell cycle in MPP^+^-treated SH-SY5Y cells, it was evaluated using flow cytometry. The cell cycle analysis showed that a sub-G_1_ apoptotic peak appeared before the G_1_ phase in SH-SY5Y cells exposed to MPP^+^. Sal suppressed the appearance of sub-G_1_ peak in a dose-dependent manner and the Trx1 inhibitor PX-12 attenuated this protective effect of Sal (Fig. 3H).

### Sal upregulates Trx1 protein and mRNA expression in MPP^+^-treated SH-SY5Y cells

**Figure 4:**
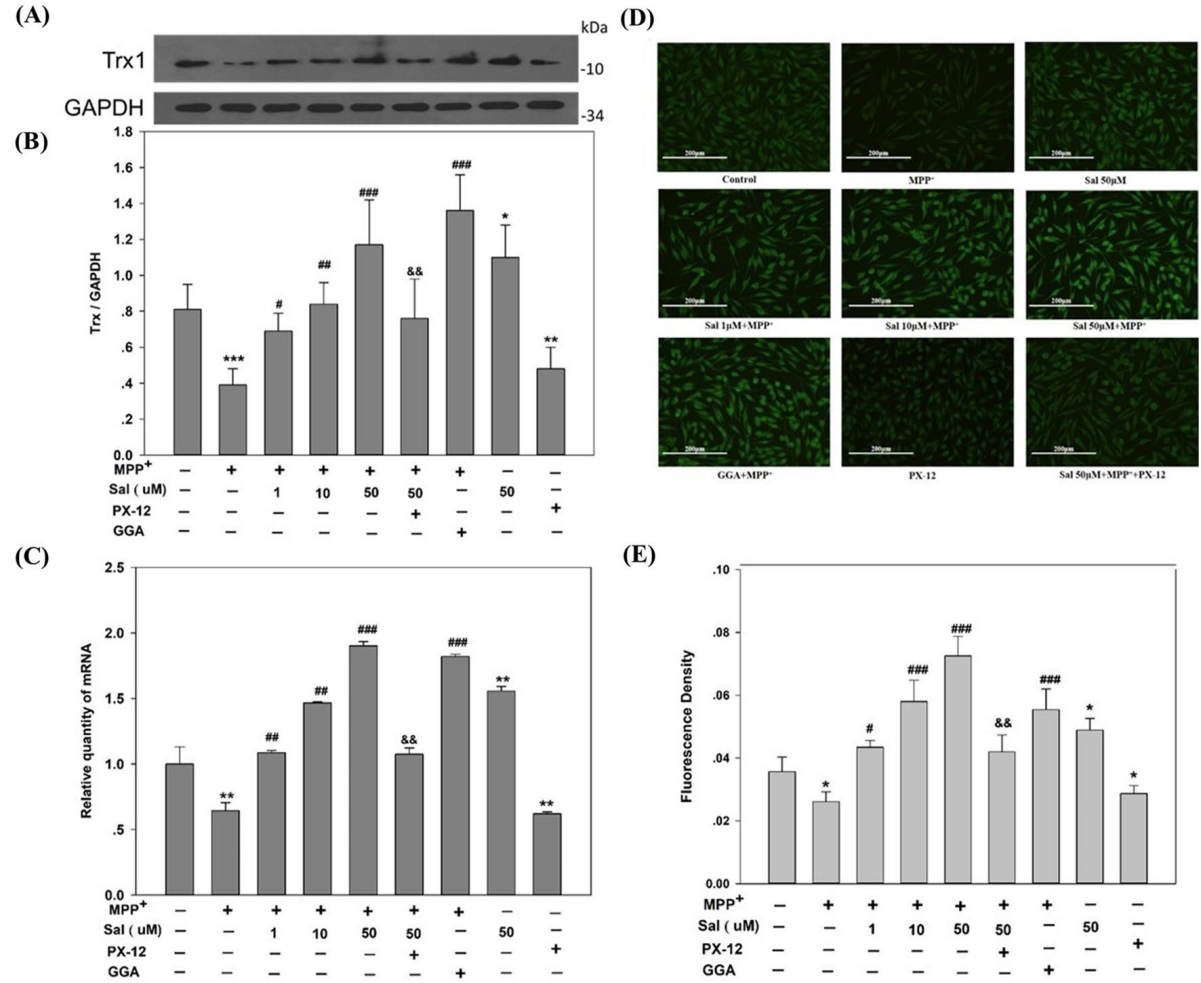
Schisanhenol increases Trx1 protein and mRNA expression in SH-SY5Y cells treated with MPP^+^. SH-SY5Y cells were treated with Sal (1 µM, 10 µM, and 50 µM) for 24 h before exposed to MPP^+^ (500 μΜ) for 48 h. (**A**) Trx1 protein levels were assayed by Western blot. A representative blot of six separate experiments was shown; (**B**) Quantification of band intensities of (**A**), Trx1 was normalized to GAPDH using Gel-Pro Analyzer 4.0 software, and the ratio of Trx1 to GAPDH was calculated; (**C**) Trx1 mRNA levels was analyzed with qPCR. Data were obtained from six separate experiments and each was repeated three times; (**D**) Trx1 expression was detected by immunofluorescence staining. The images represent the results from six independent experiments; (**E**) Quantification of Trx1 protein expression of (**D**) was performed using Image-Pro Plus 6.0. Data were expressed as mean ± SD. *P < 0.05, **P < 0.01, ***P < 0.001 vs Control group; ^#^*P* < 0.05, ^##^*P* < 0.01, ^###^*P* < 0.001 vs MPP^+^ group; ^&&^*P* < 0.01 vs Sal 50 μM + MPP^+^ group. Each experiment was conducted in triplicate.

To investigate the role of Trx1 in the protective effect of Sal on SH-SY5Y cells against MPP^+^ neurotoxicity, Trx1 expression was determined by Western blot and quantitative real-time PCR. Figure 4 shows that the application of MPP^+^ resulted in a significant decrease of Trx1 in the protein and mRNA expression levels compared to the control group (*P* < 0.05). Sal increased the Trx1 expression in the protein and mRNA levels compared to the MPP^+^ group in a dose-dependent manner. GGA also showed the same effects as Sal 50 μM, with no significant difference statistically. Compared to the control group, the Trx1 inhibitor PX-12 remarkably reduced the protein and mRNA levels of Trx1. In addition, we performed global proteomics analysis to compare protein amount change among Control, MPP^+^, and Sal 50 μM + MPP^+^ groups. The heatmap depicted the key differential proteins that we discovered in our proteomics experiment (see Supplementary Fig. S2 online). And in order to highlight proteins interacting with Trx1, the interactions of the differentially expressed proteins through network analysis using String database was deduced (see Supplementary Fig. S3 online).

### Sal regulates apoptosis-related proteins in SH-SY5Y cells exposed to MPP^+^

**Figure 5:**
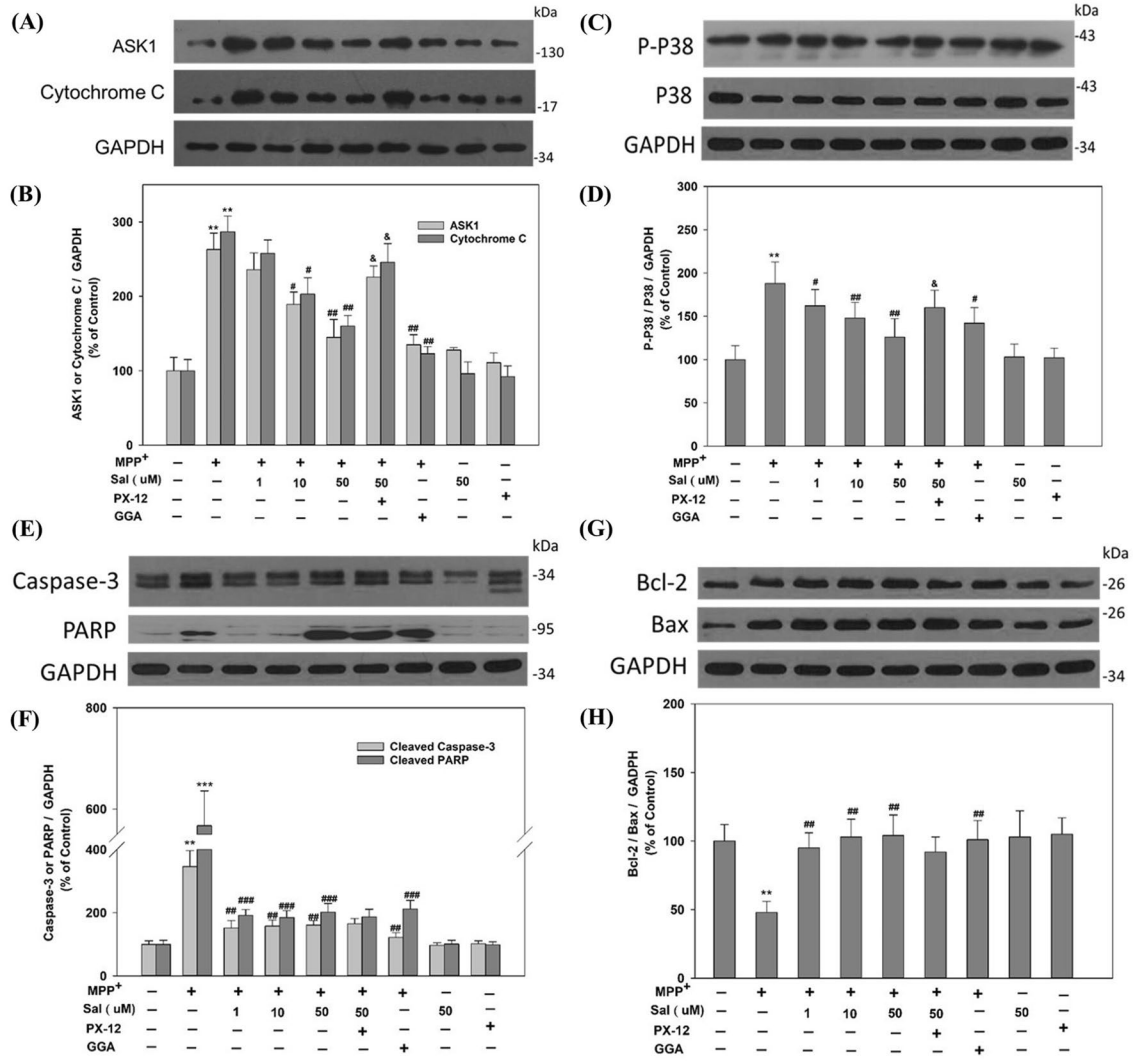
Schisanhenol decreases ASK1, cleaves Caspase-3 and PARP expression, inhibits cytochrome C release and P38 phosphorylation, and increases Bcl-2/Bax expression in SH-SY5Y cells treated with MPP^+^. Cells were treated with (500 μΜ) MPP^+^ for 48 h in the presence of Sal (1 µM, 10 µM, and 50 µM) for 24 h, and the protein levels were examined by Western blot from six separate experiments. Representative blot of ASK1 and Cytochrome C (**A**); P-P38 and P38 (C); Caspase-3 and PARP (**E**); Bcl-2 and Bax (**G**). The density of the blots was quantified, ASK1 and Cytochrome C (**B**); the ratio of P-P38/P38 (**D**); Caspase-3 and PARP (**F**); Bcl-2/Bax ratio (H). Data were expressed as mean ± SD. **P < 0.01, ***P < 0.001 vs corresponding Control group; ^#^*P* < 0.05, ^##^*P* < 0.01, ^###^*P* < 0.001 vs corresponding MPP^+^ group; ^&^*P* < 0.05 vs Sal 50 μM + MPP^+^ group. Target Protein (ASK1, Cytochrome C, etc.) levels were quantified using Gel-Pro Analyzer 4.0 software and expressed relative to GAPDH protein levels.

To explore the underlying mechanism of Sal against MPP^+^-induced cell apoptosis, the expression of proteins related to cell apoptosis was observed by Western blot. Figure 5 shows that the expression levels of apoptosis signal-regulating kinase 1 (ASK1), cleaved Caspase-3, and poly ADP-ribose polymerase (PARP) in SH-SY5Y cells were increased after treatment with MPP^+^ for 48 h. The Cytochrome C release and P38 phosphorylation (P-P38) were also promoted significantly, simultaneously with the ratio of Bcl-2/Bax decreased. However, pretreatment with Sal reduced the protein levels of ASK1, cleaved Caspase-3, and PARP but increased the Bcl-2/Bax expression. The expression level of P-P38 and the ratio of P-P38 (P-P38/P38) were also decreased compared to the MPP^+^ group, as well as Cytochrome C. The above mentioned effects of Sal on ASK1, Cytochrome C and P-P38 were attenuated by PX-12.

### Sal inhibits NF-κB activation and IκB degradation induced by MPP^+^ in SH-SY5Y cells

**Figure 6:**
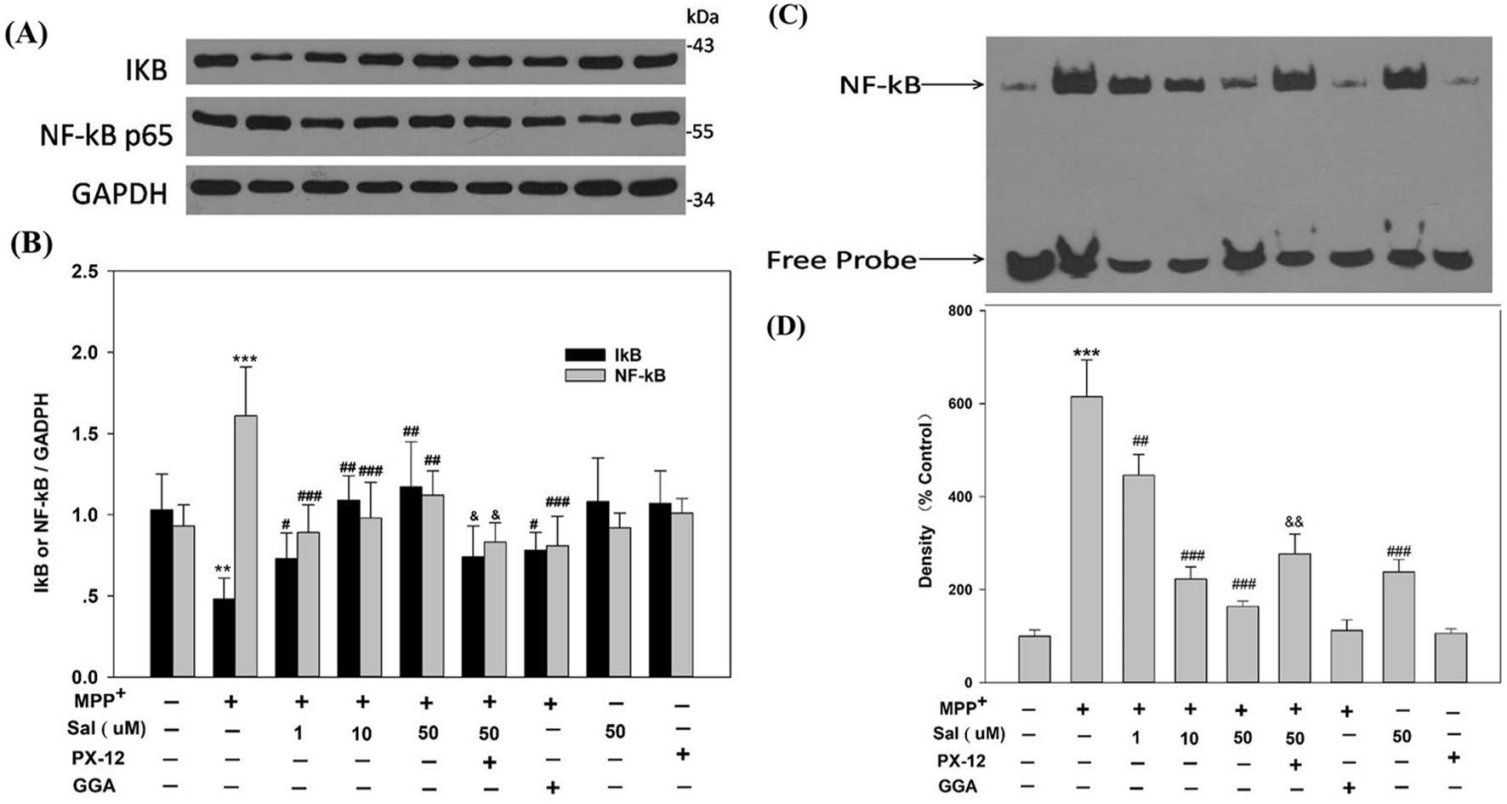
Schisanheno inhibits IκB degradation, NF-κB translocation and NF-κB-DNA binding activity in SH-SY5Y cells treated with MPP^+^ via Trx1. Cells were treated with Sal (1 µM, 10 µM, and 50 µM ) for 24 h before exposed to MPP^+^ (500 μΜ) for 48 h. The degradation of IκB and NF-κB nuclear translocation were detected by Western blot (**A** and **B**). Nuclear proteins were extracted, then NF-κB-DNA binding activity was determined by EMSA (**C** and **D**). Data were expressed as mean ± SD. **P < 0.01, ***P < 0.001 vs corresponding Control group; ^#^*P* < 0.05, ^##^*P* < 0.01, ^###^*P* < 0.001 vs corresponding MPP^+^ group; ^&^*P* < 0.05, ^&&^P < 0.01 vs corresponding Sal 50 μM + MPP^+^ group. Similar results were obtained with six separate experiments.

In order to explore the mechanism underlying the anti-apoptotic effect of Sal against MPP^+^, we detected the NF-κB translocation and IκB degradation in SH-SY5Y cells. As shown in Fig. 6A, B, the protein levels of IκB decreased in MPP^+^-treated cells, whereas pretreatment with Sal increased the protein levels of IκB. MPP^+^ induced a clear NF-κB nuclear translocation, which was inhibited by Sal pretreatment in SH-SY5Y cells. EMSA (Electrophoretic Mobility Shift Assay) results showed that pretreatment with Sal for 24 h also decreased the NF-κB-DNA binding activity induced by MPP^+^ in SH-SY5Y cells (Fig. 6C, D). These results indicated that Sal could suppress the degradation of IκB, prevent the translocation of NF-κB, and decrease the NF-κB-DNA binding activity in MPP^+^-challenged SH-SY5Y cells. The Trx1 inhibitor PX-12 attenuated the above effects of Sal.

## Discussion

The present study demonstrated that Sal, an active component from a traditional Chinese herb *Schisandra rubriflora* (Franch.), dose-dependently protected SH-SY5Y cells against MPP^+^-induced apoptosis, and Trx1 was involved in the neuroprotection of Sal.

Reports have shown that Sal has a potent antioxidant activity and neuroprotective effect. Sal scavenges oxygen radicals stimulated by tetradecanoylphorbol acetate in human neutrophils^16^, and attenuates ox-LDL-induced apoptosis and ROS generation in bovine aorta endothelial cells^20^. In the present study, MTT assay and morphological analysis showed that Sal dose-dependently prevented SH-SY5Y cells injury from MPP^+^ toxicity. And, the results of the Hoechst 33,342 staining assay, the nuclear morphological analysis, and Annexin V-FITC/PI staining by flow cytometric analysis, cell cycle, and DNA fragmentation, also demonstrated that the neuroprotection of Sal at least partly comes from its anti-apoptotic effects.

Trx1 is a redox-active protein and plays an important role in the pathogenesis of PD. Accumulating evidence has indicated that Trx1 exhibits neuroprotective effects on neurons when exposed to PD-inducing toxicants. It has been reported that overexpression or administration of Trx1 attenuated MPP^+^ -induced neurotoxicity in rat PC12 cells^12^. In the present study, we examined the expression of Trx1 in MPP^+^-treated SH-SY5Y cells with or without Sal using real time qPCR and Western blot. The results showed that both the protein and mRNA levels of Trx1 were decreased in the cells treated with MPP^+^; while these decreases were effectively prevented by Sal treatment. Moreover, we also found that the effect of Sal on Trx1 was diminished by co-treatment with Trx1 inhibitor PX-12. These results indicate that Trx1 was involved in the protective effect of Sal against MPP^+^-induced apoptosis in SH-SY5Y cells. GGA is a Trx1 inducer and has a protective agent against MPP^+^^12,24^. In the present study, the protective effect of Sal (50 μM) was similar to that of GGA. To further explore the possible molecular mechanisms of the neuroprotection of Sal, we examined the effect of Sal on apoptosis-related protein expressions in SH-SY5Y cells treated with MPP^+^. Bcl-2 family plays a key role in the mitochondrial apoptotic pathway, wherein Bcl-2 is an anti-apoptotic protein, whereas Bax exhibits pro-apoptotic activity^27^. Bax can homodimerize and activate the terminal caspases by altering the mitochondrial functions after translocation through the mitochondrial membrane; this process results in the release of apoptosis-promoting factors into the cytoplasm^28^. The Bcl-2/Bax ratio is commonly used to predict the apoptotic fate of the cell^29,30^. In the present study, we found that Sal significantly increased the Bcl-2/Bax ratio in MPP^+^-treated SH-SY5Y cells. The release of Cytochrome C from the mitochondria to the cytosol is essential to activate Caspase-3 and its downstream cell death pathways^31^. The activity of Caspase-3 is also believed to be important for the commitment or execution of neuronal apoptosis^32^. Our results showed that Sal inhibited MPP^+^-induced Cytochrome C release and decreased the activity of Caspase-3. Taken together, these results suggest that restoring the balance between positive and negative regulators of apoptosis might be one of the mechanisms of Sal’s neuroprotective action.

Caspase-3 and PARP cleavage are crucial in the cell apoptosis process, followed by activation and DNA fragmentation^33,34^. In the present study, the immunoblot analysis showed that MPP^+^ promote Caspase-3 and PARP cleavage in SH-SY5Y cells, which were significantly inhibited by Sal treatment. These findings suggest that Sal executes protection against MPP^+^-induced DNA damage and cytotoxicity.

The P38 signaling pathway exerts a critical role in cell proliferation, differentiation, survival, and apoptosis^35^. P38 enhances the cell apoptosis by direct or indirect interaction with apoptosis-related proteins, such as anti-apoptotic factor Bcl-2, pro-apoptotic factors Bax and Caspase family. Activation of P38 leads to cell death via promoting the translocation of Bax to mitochondria and activating Caspase-9 and Caspase-3^36^. In the present study, P38 was activated by exposure to MPP^+^, which appears to contribute to cell apoptosis in SH-SY5Y cells, whereas Sal suppressed the protein levels of P-P38 and the ratio of P-P38/P38 in a dose-dependent manner. ASK1 is identified as an upstream kinase of the stress response MAP kinases P38^37^, which is essential in promoting oxidative stress-induced apoptosis^38^. Similar to P38, ASK1 is also activated by oxidative stress and mediates a wide range of cell responses to oxidative stress^37,39^. Inhibition of ASK1 can abolish P38 phosphorylation and attenuate oxidative cell injury^38,40,41^. In the present study, Sal reversed the increased protein level of ASK1 by MPP^+^ in SH-SY5Y cells. These results support that Sal protects SH-SY5Y cells via regulation of apoptosis-related factors.

Oxidative damage, a common node between the cause and result of many diseases, induces multiple kinases and transcription factors in a variety of signaling pathways that promote apoptosis^42,43^. As forementioned, in the present study, Sal upregulates Trx1 expression in the protein and mRNA levels. Trx1 has a catalytic antioxidant property because its redox-active center (Cys-Gly-Pro-Cys) can reduce oxidized cysteine groups on proteins to scavenge ROS^44^. Trx1 suppresses ASK1 activity and promotes ASK1 degradation through directly binding to the N-terminal region of ASK1^39,41,45^. Under oxidative stress conditions, oxidized Trx1 is dissociated from ASK1^45^, which switches the state of ASK1 from inactive to active and generates a phosphorylation-dependent signal. ASK1 is the upstream of P38 and known to induce Caspase-3 activation and apoptosis^46^. P38 plays important roles in various cellular stress responses, including cell death, which is roughly categorized into apoptosis and necrosis. In the present study, MPP^+^ promotes apoptosis via up-regulation of the ASK1-P38 signaling pathway, while Sal prevented the apoptosis by upregulating Trx1, further lowering the ASK1 expression and suppressing P38 phosphorylation.

NF-κB signaling pathway is involved in many neurodegenerative diseases, such as Alzheimer’s disease and PD. NF-κB belongs to the Rel transcription factor family, regulating the expression of apoptosis-related genes and controlling cell division and apoptosis^47^. Moreover, it has been reported that nuclear translocation of NF-κB increased in dopaminergic neurons of PD patients^48^. When NF-κB is activated and translocated to the nuclei, this transcriptional factor binds to those response elements and triggers target genes involved in cell death. To investigate the role of NF-κB pathway in the protective effect of Sal on MPP^+^-induced apoptosis, we investigated degradation of IkB and translocation of NF-κB using Western blot analysis, and NF-κB-DNA binding activity by EMSA. We found that the degradation of IkB, nuclear translocation of NF-κB, and DNA binding activity were all enhanced after MPP^+^ treatment, which consists with the results of reported studies^49,50^. And, we also found that Sal pretreatment decreased the nuclear NF-κB level and NF-κB-DNA binding activity, as well as increased the IkB expression in a dose-dependent manner. These results suggest that Sal protects SH-SY5Y cells from MPP^+^ toxicity by inhibiting NF-κB signaling pathway.

In order to boost significance of our findings, we performed global proteomics analysis to compare protein amount change among Control, MPP^+^, and Sal 50 μM + MPP^+^ groups. The heatmap depicted the key differential proteins that we discovered in our proteomics experiment. Meanwhile, we were able to deduce some information from the GO annotation and updated STRING Interaction analysis in order to highlight proteins interacting with Trx1. As shown in Fig. S3, with the addition of MPP^+^ substances, the protein abundance of GO term (negative regulation of oxidative stress-induced neuron death, response to oxidative stress) was considerably enhanced in the samples. Furthermore, the expression of protein COP9 in the NF-κB pathway, which is a source of concern for us, was reduced, which is exactly what we expected for both of them. Furthermore, the MPP^+^ group exhibited a higher protein abundance of the mRNA splicing pathway, which may necessitate additional investigation in the future.

**Figure 7:**
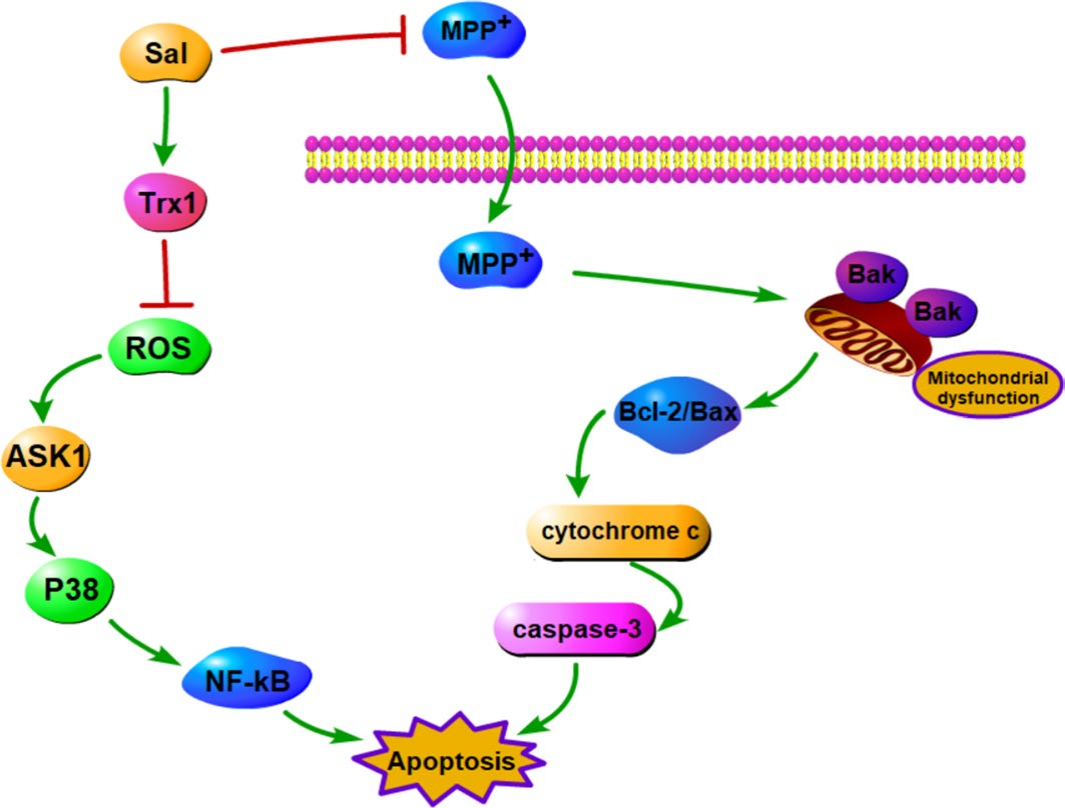
Schisanhenol protects against MPP^+^-induced apoptosis in SH-SY5Y cells via up-regulation of Trx1 expression, subsequently suppression of ASK1-P38-NF-κB pathway.

In conclusion, this study has demonstrated that Sal exerts protective effects against MPP^+^-induced apoptotic damage in SH-SY5Y cells through Trx1-mediated suppression of the ASK1-P38-NF-κB signaling pathway (summarized in Fig. 7). Our results also suggest that Sal might be a promising neuroprotective agent for prevention of PD.

## Materials and methods

### Chemicals and reagents

Sal [99% purity based on high-performance liquid chromatography (HPLC)] was purchased from Chengdu Push Bio Technology Co., Ltd. (Chengdu, China). MPP^+^, MTT, and Hoechst 33,342 were provided by Sigma-Aldrich (St. Louis, MO, USA). PX-12 was purchased from Tocris Bioscience (Bristol, UK). GGA was from Tokyo Kasei Kogyo Co., Ltd. (TCI) (Tokyo, Japan). Dulbecco’s modified Eagle’s medium/F-12 medium (DMEM/F-12) and fetal bovine serum were obtained from Thermo Scientific HyClone (Logan, UT, USA). The primary antibody against Trx1 was purchased from Abcam (Cambridge, UK); antibodies against Cytochrome c, Bcl-2, and Bax were from Santa Cruz Biotechnology (Santa Cruz, CA, USA); and antibodies against ASK1, Caspase-3, P38, phospho-P38, PARP, NF-κB, IκB, GAPDH, and horseradish peroxidase (HRP)-conjugated secondary antibody were obtained from Cell Signaling Technology (Beverly, MA, USA). All other chemicals and reagents were of analytic grade.

### Cell culture and treatment

Human neuroblastoma SH-SY5Y cells were purchased from the Cell Culture Center, Institute of Basic Medical Sciences of Chinese Academy of Medical Sciences (Beijing, China). Cells were cultured in a 1:1 mixture of DMEM and F-12 medium containing 10% fetal bovine serum, supplemented with 100 U/mL penicillin and 100 μg/mL streptomycin (Invitrogen, Carlsbad, CA, USA), in an incubator with humidified atmosphere of 5% CO_2_ at 37 °C. Cells were incubated with different doses of Sal or co-treated with GGA (10 μM) or PX-12 (5 μM) for 24 h before exposure to MPP^+^ for 48 h. All experiments were conducted in compliance with the Cell Experiment Guidelines of China.

### Cell viability assay

Cell viability was determined by MTT assay. SH-SY5Y cells were seeded into a 96-well plate (1 × 10^4^ cells per well). After Sal and MPP^+^ treatment, MTT was added with a final concentration of 0.5 mg/mL; the cells were incubated at 37 °C for 4 h. Then, the medium was removed gently, and 150 μL of DMSO was added; the mixture was then shaken for 10 min to dissolve the formazan crystals. The absorbance at 570 nm was measured using a microtiter plate reader (Tecan, Männedorf, Switzerland). Cell viability was described as a percentage compared with the control group.

### Morphological observation

SH-SY5Y cells were seeded into a 96-well plate (1 × 10^4^ cells per well). After treatment, the morphology of SH-SY5Y cells was observed under an inverted phase contrast microscope (Olympus, IX 51, Tokyo, Japan).

### Western blot

Cells were collected after treatment, and the total protein was extracted in a cell lysis buffer (Beyotime Institute of Biotechnology, Shanghai, China). After the protein concentration was determined using the BCA protein assay kit (Beyotime Institute of Biotechnology, Shanghai, China), equal amounts of protein samples were electrophoresed on a sodium dodecyl sulfate–polyacrylamide gel (SDS-PAGE) (12% (w/v) polyacrylamide gel) and transferred to a polyvinylidene fluoride (PVDF) membranes (Pall Corp., Port Washington, NY, USA). Thereafter, the membranes was blocked with 5% non-fat milk in Tris-buffered saline containing 0.1% Tween-20 (TBST) for 1 h at room temperature. This process was followed by an overnight incubation with corresponding primary antibodies in TBST at 4 °C. The membranes were then washed thrice in TBST buffer, followed by incubation with 1:2000 dilutions of HRP-conjugated secondary antibody for 1.5 h at room temperature. The membranes were washed again three times in TBST. The proteins were visualized using the enhanced chemiluminescence (ECL) plus kit (Applygen Technologies Inc., Beijing, China). The membranes were then scanned using a Bio-Rad ChemiDoc XRS imaging system; the density of the bands on the blots was quantified by Gel-Pro Analyzer 4.0 software (Media Cybernetics, Rockville, MD, USA).

### RNA isolation and real-time quantitative polymerase chain reaction (qPCR)

After treatment, SH-SY5Y cells were harvested and the total RNA was extracted using the RNAiso reagent kit (Takara Biotechnology, Dalian, China). The cDNA was synthesized with an ExScript RT kit based on the manufacturer’s instructions. Measurement of mRNA levels was performed using an ABI 7300 real-time qPCR system (Applied Biosystems, Foster City, CA, USA) using SYBR Premix Ex Taq (Takara Biotechnology, Dalian, China). In real-time qPCR amplification, primer sequences were used as follows: Trx1, sense primer, 5′-ATCAAGCCTTTCTTTCATTCCCTCT-3′, antisense primer, 5′- TTCACCCACCTTTTGTCCCTTC-3′; β-actin, sense primer, 5′- ACTTAGTTGCGTTACACCCTT-3′, antisense primer, 5′- GTCACCTTCACCGTTCCA-3′. All primers were purchased from Takara Biotechnology (Dalian, China). The thermal cycling parameters were used as follows: 95 °C for 30 s, 40 cycles at 95 °C for 5 s, and 60 °C for 31 s. The relative quantitative values of Trx1 expression were normalized to β-actin gene. Threshold cycle (Ct) data were collected using Sequence Detection Software version 1.2.3 (Applied Bio-systems). Relative quantification of gene expression was analyzed using the 2^−ΔΔ^Ct method. Ct values of the Trx1 genes were normalized to the Ct values of β-actin to obtain delta Ct (ΔCt). Fold change in Trx1 genes relative to the β-actin internal control was determined by the following equation: Fold change = 2^−ΔΔCt^, where ΔΔCt = (Ct_Trx1_ − Ct_β-actin_) − (Ct_control_ − Ct_β-actin_).

### Immunofluorescence staining

Cells were fixed with 95% ethanol, washed with phosphate-buffered saline (PBS) three times, blocked in non-immune goat serum at 37 °C for 30 min, and incubated with primary Trx1 antibody (1: 200, Abcam, Cambridge, UK) at 4 °C overnight. After washing thrice with PBS, the sections were incubated with fluorescein isothiocyanate (FITC) -conjugated secondary antibodies, goat anti-rabbit IgG (Cowin Biotech Co., Ltd., Beijing, China), at a 1: 25 dilution at 37 °C for 1 h. Cell images were obtained using an inverted fluorescence microscope (Carl Zeiss, Axiovert 200, Oberkochen, Germany), and analyzed using Image-Pro Plus 6.0 (Media Cybernetics, Inc., Rockville, MD, USA).

### Annexin V-FITC/PI staining

Apoptosis was evaluated using an Annexin V-FITC/PI apoptosis detection kit (Nanjing KeyGEN Biotech. CO. LTD., Nanjing, China) according to the manufacturer’s instruction. After treatments, approximately 1 × 10^5^ cells from each group were harvested and washed twice with PBS. Then, the cells were resuspended in 500 μL binding buffer; 5 μL of Annexin V-FITC and 5 μL of PI were subsequently added. After gentle vortexing, all the cells were incubated at room temperature in the dark for 10 min. Thereafter, the cells were subjected to flourescence-activated cell sorting (FACS) analysis (BD Biosciences, San Jose, CA, USA) within 1 h. Flow cytometry was used to count the number of cells that underwent apoptosis, and the percentages in the total number of cells in each group were compared. Finally, the staining cells were placed on the glass slides, and images were obtained under a fluorescence microscope (Carl Zeiss, Axiovert 200, Oberkochen, Germany). Cell analysis was performed using Image-Pro Plus 6.0 (Media Cybernetics, Inc., Rockville, MD, USA).

### Caspase-3 activity assay

The activity of Caspase-3 was determined using Caspase-3 colorimetric assay kit (BioVision, Inc., Milpitas, CA, USA) based on the manufacturer’s protocol. The cells were prepared in lysis buffer and centrifuged at 10,000 g for 1 min. Appropriate dilution was prepared following the protein concentration assay. Each sample was added with a reaction buffer (containing DTT) and DEVD-pNA substrate before incubated at 37 °C for 1.5 h. Then, the samples were detected at 405 nm using a microtiter plate reader. Fold-increase in Caspase-3 activity was determined by comparing these results with the level of the control group. Background reading from cell lysates and buffers had been subtracted from the readings of both induced and non-induced samples before calculating the fold increase in the Caspase-3 activity.

### Hoechst 33,342 staining

SH-SY5Y cells were seeded into 12-well plates (8 × 10^4^ cells per well). After treatment, the cells were washed with PBS, and fixed with 4% formaldehyde (v/v in PBS) before stained with Hoechst 33,342 (10 μg/mL) for 15 min at room temperature. After washed with PBS twice, images of the nuclei were obtained using a fluorescence microscope equipped with a camera (Carl Zeiss, AxioCam HRc, Oberkochen, Germany) at 460 nm.

### Cell cycle analysis

The influence of Sal on cell cycle was detected using BD Cycletest Plus DNA Reagent Kit (BD Biosciences, San Jose, CA, USA) based on the manufacturer’s instructions. At the end of the treatment, the cells were washed, centrifuged, and resuspended in the buffer solution for staining and flow cytometric analysis. A total of 5 × 10^5^ cells were added trypsin buffer to react for 10 min, as well as solution containing trypsin inhibitor and RNase buffer to incubate for 10 min at room temperature. Thereafter, propidium iodide (PI) staining solution was added in the dark on ice to incubate for further 10 min. Following filtration using a 50 μm nylon mesh, the samples were run on the flow cytometer by BD FACSCalibur Flow Cytometry System (BD Biosciences, San Jose, CA, USA). Data were analyzed with ModFit LT software (Verity Soft-ware House, Topsham, ME, USA).

### DNA fragmentation analysis by agarose gel electrophoresis

DNA was extracted using Genomic DNA Mini Preparation Kit with Spin Column (Beyotime Institute of Biotechnology, Shanghai, China). After treatment, the cells were collected and resuspended in PBS, proteinase-K and lysis buffer were subsequently added, and incubated at 70 °C for 10 min. Subsequently, 100% ethanol was added, and the mixture was transferred to the purifying column to obtain DNA after centrifugation and elution. The pelleted DNA was dissolved in TE buffer, electrophoresed on 1.5% agarose gel (Takara Biotechnology, Dalian, China) pre-stained with 0.5 g/mL ethidium bromide (EB) at 50 V for 2 h. The DNA ladders were finally visualized by a UV light source and documented by photography.

### Electrophoretic Mobility Shift Assay (EMSA)

Nuclear factor kappa B (NF-κB)-DNA binding activity was determined by EMSA. Nuclear extracts from the treated SH-SY5Y cells were prepared using NucBuster Protein Extraction Kit (Merck Millipore, Billerica, Massachusetts, USA). 5’-Biotin-labeled double-stranded NF-κB oligonucleotides (5’-AGTTGAGGGGACTTTCCCAGGC-3’, Invitrogen, Carlsbad, CA, USA) were used as probe. EMSA was performed using a LightShift™ Chemiluminescent EMSA Kit (Thermo Scientific, Waltham, MA, USA) following the manufacturer’s protocol. 5 μg of the nuclear protein extract was mixed with 30 pM of biotin-labeled NF-κB DNA probe in binding buffer (10 mM Tris, pH7.5, 50 mM KCl, 1 mM dithiothreitol, 1 μg/μL poly (dI-dC)) for 20 min at room temperature. Competitive reactions were performed by adding 200-fold molar excess of unlabeled oligonucleotides. DNA–protein complexes were separated on a 6% non-denaturing polyacrylamide gel with 0.5 × TBE buffer, transferred to a nylon membrane, and visualized using the chemiluminescent nucleic acid detection module provided in the EMSA kit.

### Statistical analysis

Data were expressed as mean ± SD. Statistical analysis were conducted with SPSS 15.0 software (SPSS Inc., Chicago, IL, USA) applying one-way analysis of variance (ANOVA) followed by LSD test. *P* < 0.05 was considered statistically significant.

## Supplementary Information

Supplementary Information.
