# Supplementary material for: Thioredoxin-1 mediates neuroprotection of Schisanhenol against MPP+-induced apoptosis via suppression of ASK1-P38-NF-κB pathway in SH-SY5Y cells
Source: Sci Rep. 2021 Nov 3;11:21604. doi: 10.1038/s41598-021-01000-3 (PMC8566484; doi:10.1038/s41598-021-01000-3)
Supplement: Supplementary file 1 — Supplementary Information. [file 41598_2021_1000_MOESM1_ESM.docx]

**Thioredoxin-1 mediates neuroprotection of Schisanhenol against MPP^+^-induced apoptosis via suppression of ASK1-P38-NF-κB pathway in SH-SY5Y cells**

Hongyan Yang ^1^, Libo Li ^2,^*, Yu Jiao ^2^, Yuanliang Zhang ^3^, Yuhua Wang ^2^, Kunjie Zhu ^4^, Chao Sun ^1^

^1^Department of Pharmacy, Qiqihar Medical University, Qiqihar, China

^2^School of Mental Health, Qiqihar Medical University, Qiqihar, China

^3^State Key Laboratory of Chemical Biology and Drug Discovery, Department of Applied Biology and Chemical Technology, Hong Kong Polytechnic University, Hong Kong, China

^4^Basic Medicine School, Qiqihar Medical University, Qiqihar, China

^*^**Corresponding author**

Libo Li, School of Mental Health, Qiqihar Medical University, 333 Bukui Street, Jianhua District, Qiqihar 161006, China.

E-mail: [liliboqqhr@163.com](mailto:liliboqqhr@163.com); Tel: +86-452-266 3831


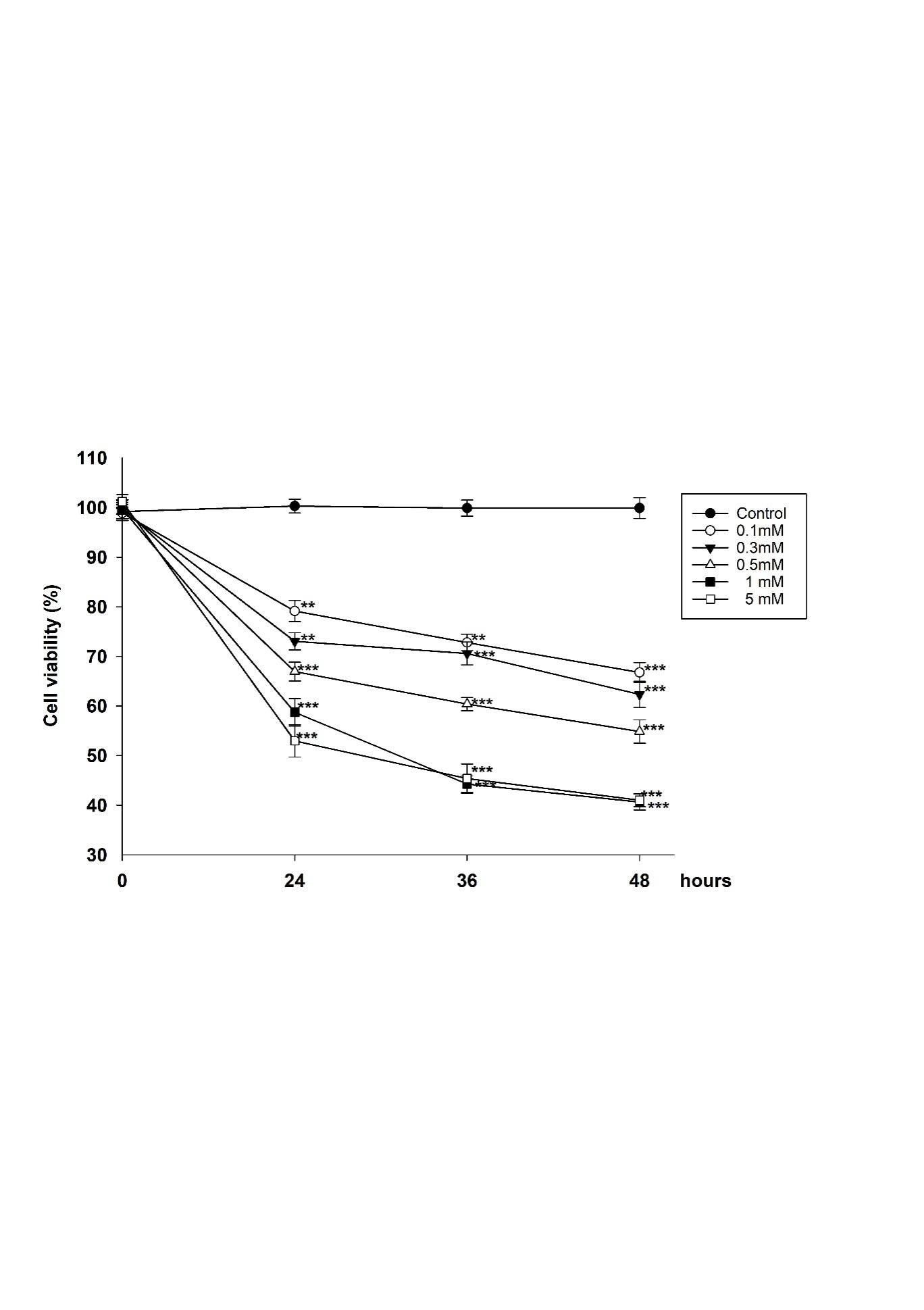


**Figure S1.** Time- and dose-effect of MPP^+^ on viability of SH-SY5Y cells. After cell exposure to various concentrations of MPP^+^ (0.1 mM, 0.3 mM, 0.5 mM, 1 mM, and 5 mM) for different time (24 h, 36 h, and 48 h), cell viability was measured using the MTT assay with six separate experiments. Each experiment was conducted in triplicate. Data were expressed as mean ± SD. **P<0.01, ***P<0.001 vs Control group.


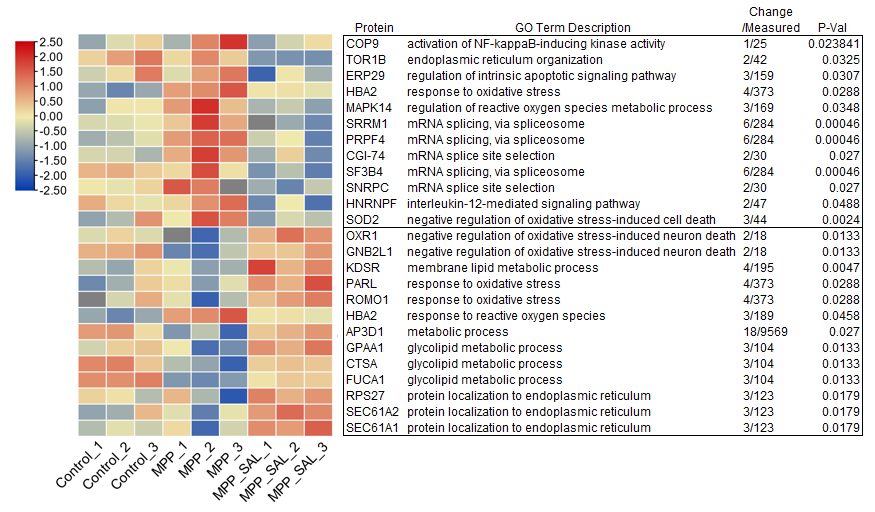


**Figure S2.** Heatmap visualization and GO annotation of key different expressed proteins between MPP^+^ and Sal/MPP^+^ group. Control, MPP+, and Sal 50 μM / MPP^+^ with triplicate biological replication.


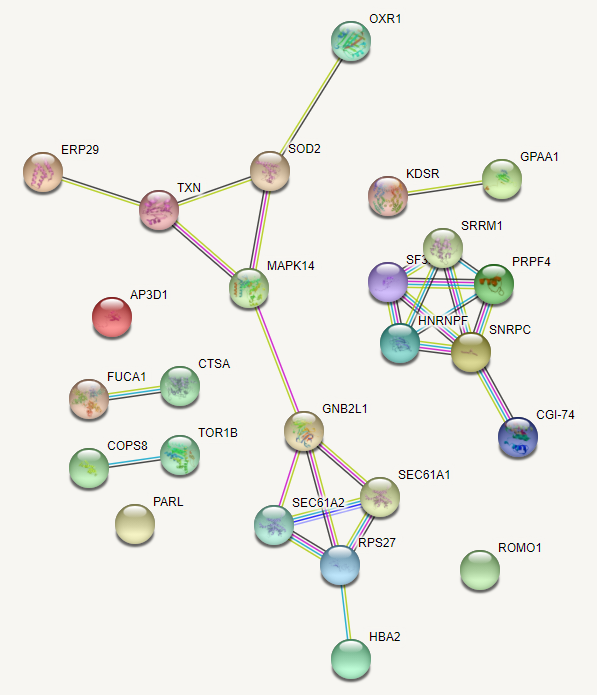

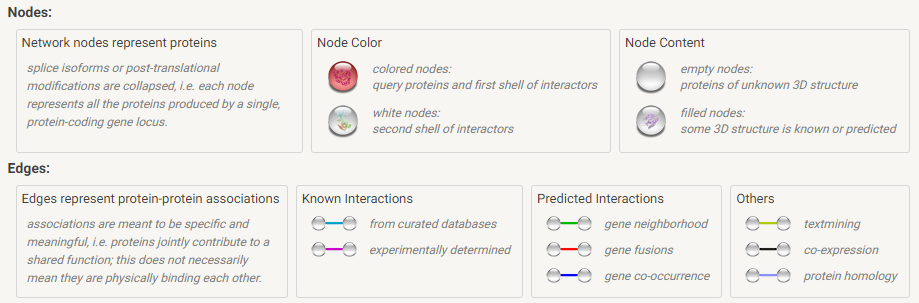


**Figure S3.** Interactions of the differentially expressed proteins through network analysis using String database.

**Fig.4A**


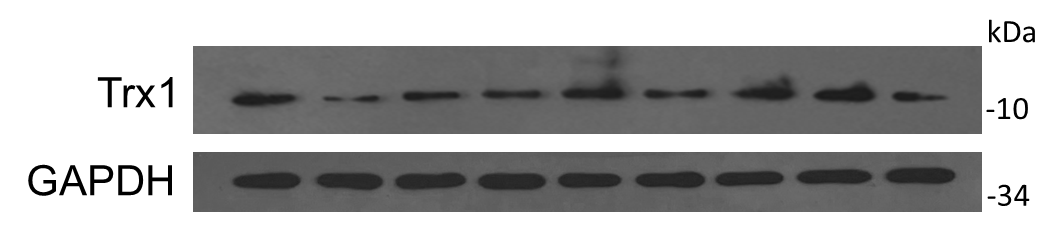


**Original blots for Fig.4A**

**Trx1**


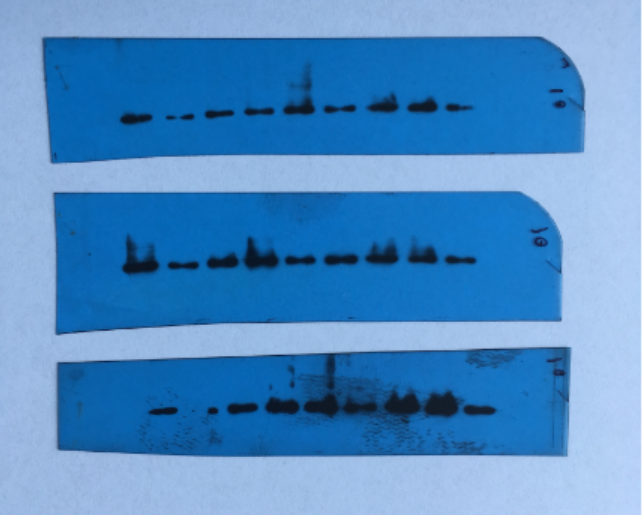


**GAPDH for Trx1，ASK1 and Cytochrome C**


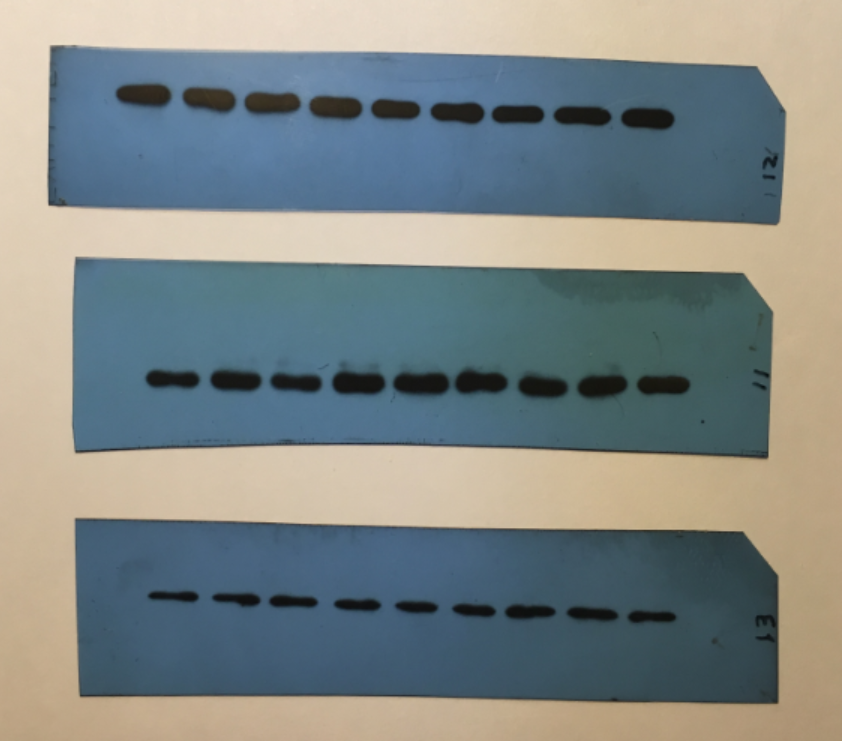


**Fig.5A**


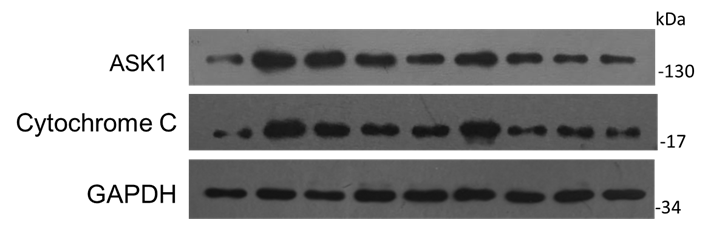


**Original blots for Fig.5A**

**ASK1**


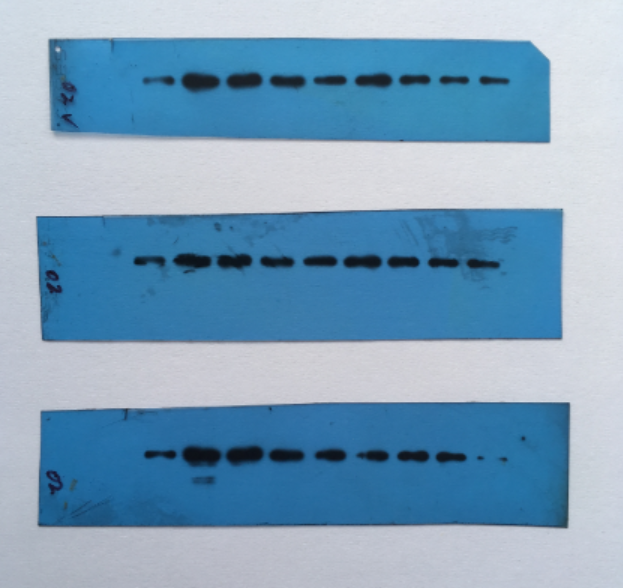


**Cytochrome C**


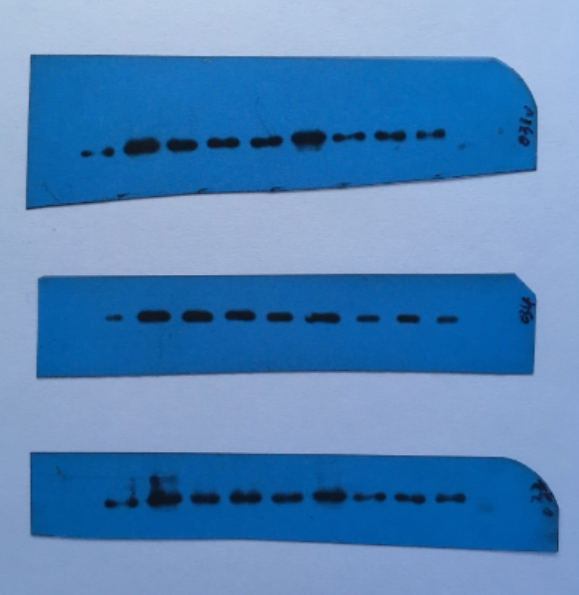


**Fig.5C**


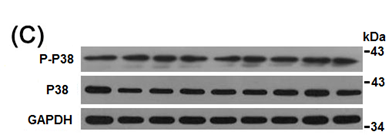


**Original blots for Fig.5C**


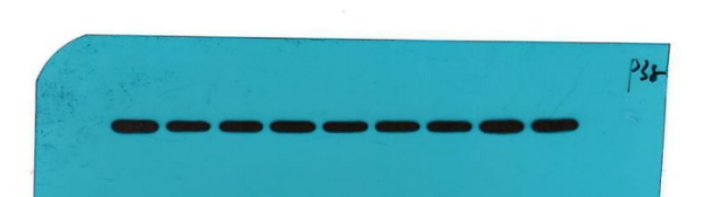

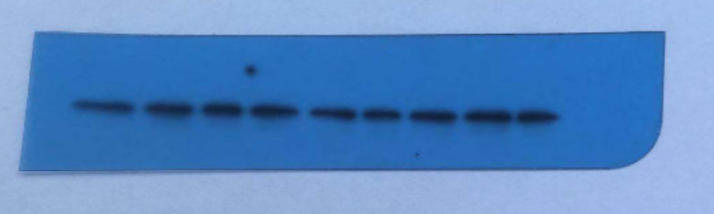


**P-P38**


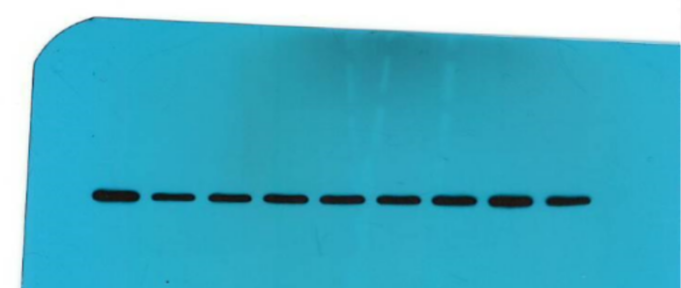


**GAPDH for P-P38 and P38**

**P38**

**Fig.5E**


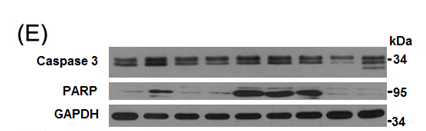


**Original blots for Fig.5E**


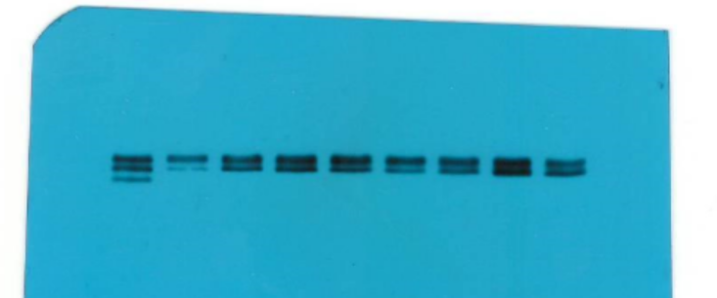


**Caspase 3**


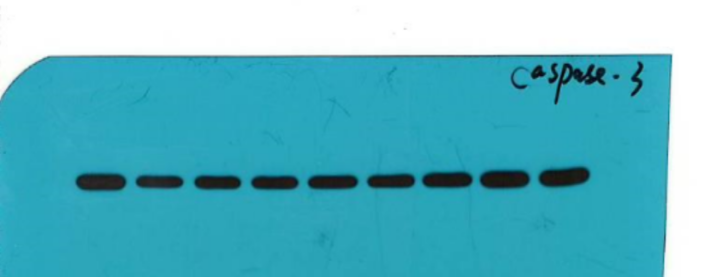

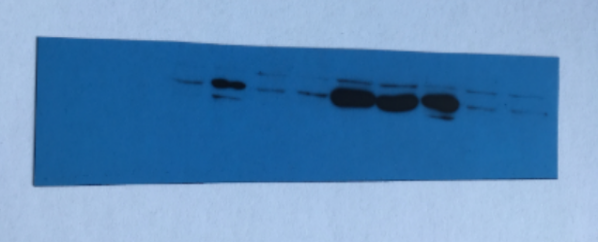


**PARP**

**GAPDH for Caspase 3 and PARP**

**Fig.5G**


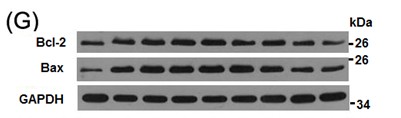


**Original blots for Fig.5G**


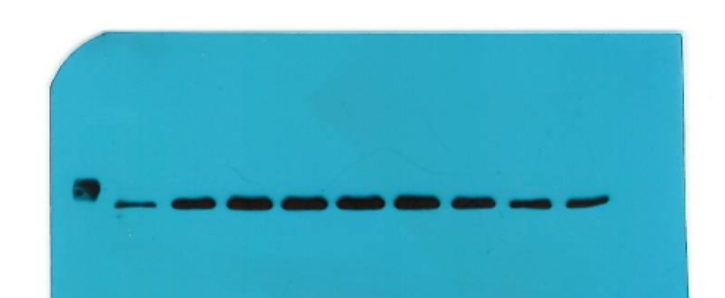

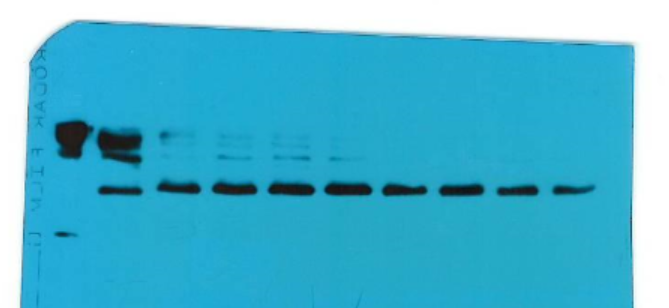


1. **Bax**
2. **Bcl-2**


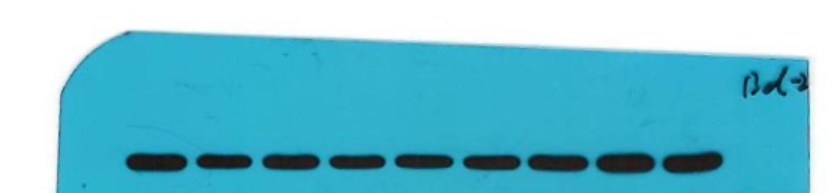


1. **GAPDH for Bcl-2 and Bax**

**Fig.6A**


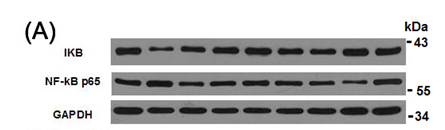


**Original blots for Fig.6A**


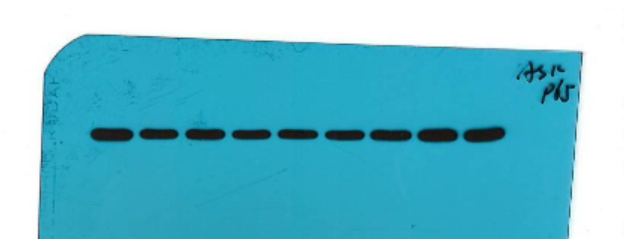


**IκB**


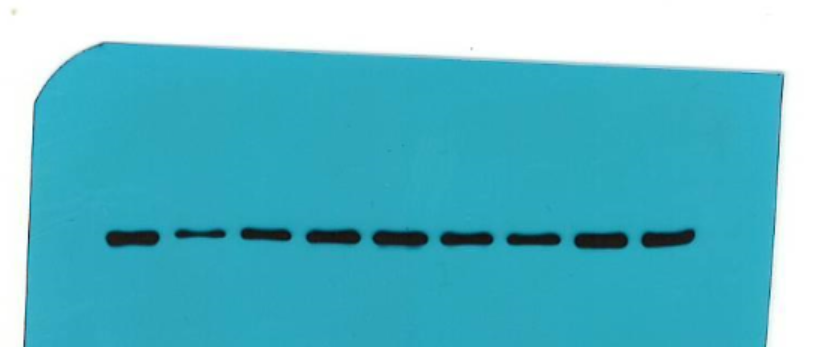


**NF-κB P65**


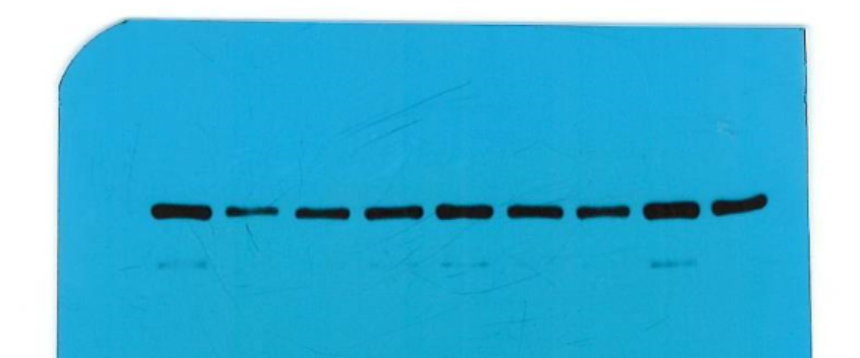


**GAPDH for IκB and NF-κB P65**
